# Blocking ADAM17 Function with a Monoclonal Antibody Improves Sepsis Survival in a Murine Model of Polymicrobial Sepsis

**DOI:** 10.3390/ijms21186688

**Published:** 2020-09-12

**Authors:** Hemant K. Mishra, Jing Ma, Daniel Mendez, Robert Hullsiek, Nabendu Pore, Bruce Walcheck

**Affiliations:** 1 Department of Veterinary and Biomedical Sciences, University of Minnesota, St. Paul, MN 55108, USA; hkumar@umn.edu (H.K.M.); maxxx221@umn.edu (J.M.); dmendez@umn.edu (D.M.); hulls015@umn.edu (R.H.); 2 Early Oncology Clinical Science, Astra Zeneca, One Medimmune Way, Gaithersburg, MD 20878, USA; nabendu.pore@astrazeneca.com

**Keywords:** sepsis, ADAM17, inflammation, immunosuppression, neutrophil

## Abstract

Sepsis is the culmination of hyperinflammation and immune suppression in response to severe infection. Neutrophils are critical early responders to bacterial infection but can become highly dysfunctional during sepsis and other inflammatory disorders. The transmembrane protease ADAM17 (a disintegrin and metalloproteinase 17) is expressed by leukocytes and most other cells and has many substrates that regulate inflammation. We have reported that conditional knockout mice lacking ADAM17 in all leukocytes had a survival advantage during sepsis, which was associated with improved neutrophil effector functions. These and other findings indicate aberrant ADAM17 activity during sepsis. For this study, we evaluated for the first time the effects of an ADAM17 function blocking monoclonal antibody (mAb) on the pathogenesis of polymicrobial sepsis. Mice treated with the ADAM17 mAb MEDI3622 prior to sepsis induction exhibited significantly decreased mortality. When the ADAM17 mAb was combined with antibiotic administration, sepsis survival was markedly enhanced compared to either intervention alone, which was associated with a significant reduction in plasma levels of various inflammation-related factors. MEDI3622 and antibiotic administration after sepsis induction also significantly improved survival. Our results indicate that the combination of blocking ADAM17 as an immune modulator and appropriate antibiotics may provide a new therapeutic avenue for sepsis treatment.

## 1. Introduction

Sepsis is a complex and heterogenous clinical syndrome caused by a highly disrupted immune response during severe infection [1]. It is a leading cause of mortality worldwide and one the most expensive conditions treated in US hospitals [2,3]. Neutrophils are the most numerous circulating leukocyte population in humans and undergo a rapid and robust influx into infected tissues [4,5]. Neutrophils must achieve a critical tissue concentration to control bacterial infection [6,7]. Neutrophils are capable of very robust recruitment to sites of infection that is facilitated by various adhesion and chemoattraction processes [4]. A number of studies have shown, however, that the highly efficient manner of neutrophil influx into sites of infection can become compromised during sepsis, a type of dysfunction that has been referred to as neutrophil paralysis [8]. Understanding the central mediators of neutrophil paralysis during sepsis and other conditions of hyperinflammation provides opportunities for new therapeutic targets.

ADAM17 (a disintegrin and metalloproteinase 17) is a transmembrane protease constitutively expressed by most cells, including leukocytes [9,10]. It is well established that cell activation by a number of physiological stimuli rapidly induces ADAM17′s proteolytic activity, which involves an increase in its intrinsic activity induced by conformational changes and intermolecular interactions [9]. Substrate cleavage by ADAM17 is referred to as ectodomain shedding and typically occurs in a *cis* manner at a single extracellular site proximal to the cell membrane [9]. ADAM17 has various substrates with diverse functions, including cytokines, cytokine and chemokine receptors, other receptors, and adhesion proteins [9,10]. ADAM17 directly regulates various neutrophil effector functions, such as their migration, their release and recognition of pro-inflammatory factors, and their phagocytosis of antibody-opsonized targets, which involves the well described substrates L-selectin (CD62L), CXCR2 (C-X-C Motif Chemokine Receptor 2), interleukin (IL)-6R, tumor necrosis factor (TNF) α, and CD16 [9,10,11,12,13]. Excessive and prolonged induction of ADAM17 appears to contribute to various inflammatory disorders [9,14]. We have reported that during sepsis, ADAM17 promotes neutrophil paralysis. For instance, mice with ADAM17-null leukocytes showed significantly improved survival during monomicrobial and polymicrobial sepsis [15,16,17], and this increased resistance corresponded with higher neutrophil recruitment at sites of infection and decreased bacterial levels locally and systemically [15,16,17]. Moreover, in patients, ADAM17 activity and a functional polymorphism of its gene corresponded with sepsis progression [18,19]. These findings implicate a critical role played by ADAM17 in the pathogenesis of sepsis and its potential as a therapeutic target.

In the current study, we investigated for the first time the in vivo effects of systemic ADAM17 inhibition using a function blocking monoclonal antibody (mAb) on sepsis resistance. MEDI3622 is a well characterized ADAM17 mAb and a highly selective and potent inhibitor [20,21]. We have shown MEDI3622 recognizes mouse as well as human leukocytes and effectively blocks ectodomain shedding of ADAM17 substrates [22]. Using a murine model of polymicrobial sepsis induced by cecal ligation and puncture (CLP), MEDI3622 was administered either pre- or post-CLP and was also used in combination with a broad-spectrum antibiotic. We found that the combined treatments markedly increased survival, neutrophil infiltration at the site of infection, and also reduced systemic cytokine levels and their proinflammatory profile. 

## 2. Results and Discussion

### 2.1. Administration of a mAb That Blocks ADAM17 Function Increases Sepsis Survival

**Figure 1:**
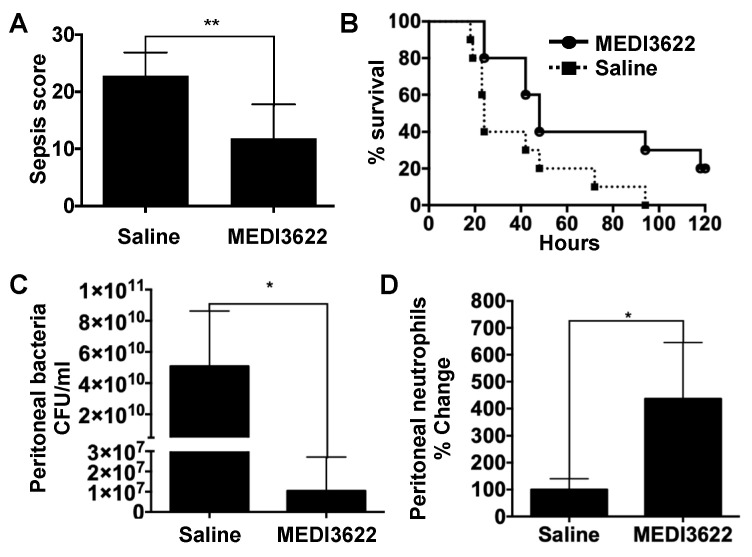
MEDI3622 administration prior to sepsis induction. (**A**) Mice were *ip* (intraperitoneally) administered MEDI3622 (15 mg/kg) or saline carrier alone (control) and 24 h later subjected to cecal ligation and puncture (CLP) (18G needle). Sepsis severity was assessed at 12 h post-CLP using a murine sepsis scoring system, as described in the Materials and Methods. *N* = 10 per group, data presented as mean ± standard deviation (SD), ** *p* < 0.01. (**B**) Survival comparison between the two treatment groups was done by a log-rank test. *N* = 10 per group, *p* < 0.05. Bacterial (**C**) and neutrophil (**D**) levels in the peritoneal cavity were determined 24 h after sepsis induction. *N* = at least 4, data presented as mean ± SD, * *p* < 0.05.

MEDI3622 is an ADAM17 function-blocking mAb that recognizes a conserved epitope occurring in human and mouse ADAM17 [20,21,22]. Twenty-four hours prior to sepsis induction, mice were administered MEDI3622 (15 mg/kg) or saline carrier intraperitoneally. Mice were then subjected to severe sepsis by CLP. Twelve hours post-CLP, a time point past potential anesthesia interference and before a moribund state, sepsis severity was evaluated by a murine sepsis scoring system of early physiologic parameters [16]. Mice treated with MEDI3622 mAb demonstrated a significantly lower sepsis score in comparison to the control group (Figure 1A), indicating reduced sepsis severity. Indeed, MEDI3622-treated mice also had a significantly improved survival rate (Figure 1B). 

Neutrophils are well established in terms of their essential role in host defense against bacterial infections [5]. In studies using ADAM17 conditional knockout mice in which all leukocytes lacked functional ADAM17, we reported that neutrophil emigration is significantly increased at the site of infection during sepsis compared to control mice [16]. Therefore, we focused on these cells in our current study as a potential immune mechanism contributing to the increased survival of MEDI3622-treated mice during sepsis. A time point of substantial contrast in sepsis susceptibility between MEDI3622-treated and control mice and at which sufficient surviving mice could be examined was at 24 h post-CLP (Figure 1B). Mice treated with MEDI3622 exhibited a significantly lower bacterial load (Figure 1C) and higher numbers of recruited neutrophils in the peritoneal cavity (Figure 1D). These results suggest that systemic ADAM17 inhibition by MEDI3622 enhanced resistance to sepsis, which corresponded with increased neutrophil recruitment and bacterial clearance at the site of infection. We cannot rule out, however, increased bacterial elimination by additionsl leukocyte populations, including other professional phagocytes such as resident macrophages and recruited monocytes. Various adhesion molecules and chemokine receptors expressed by leukocytes and endothelial cells are ADAM17 substrates and the sheddase has been directly shown to regulate intravascular adhesion events [9,12,14,23]. The leukocyte adhesion protein L-selectin, for instance, is a very well characterized ADAM17 substrate [9,11]. Its function has been reported to be instrumental in directing neutrophils into the peritoneal cavity following CLP-induced sepsis [24]. Moreover, directly disrupting the shedding of L-selectin through site-directed mutagenesis increased neutrophil recruitment into the peritoneal cavity during bacterial infection [17].

### 2.2. MEDI3622 in Combination with Antibiotic Treatment Further Enhances Sepsis Resistance

**Figure 2:**
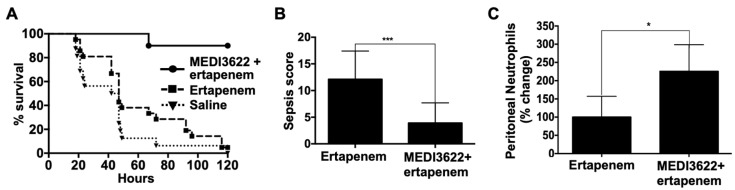
Effects of MEDI3622 and antibiotic treatment on sepsis response. (**A**) All mice were subjected to CLP (18G needle). Separate groups of mice were *ip* administered MEDI3622 (15 mg/kg) 24 h prior to sepsis and ertapenem (75 mg/kg) *ip* at 6, 24, and 48 h post-CLP; ertapenem *ip* at 6, 24, and 48 h post-CLP, or saline *ip* at 6, 24, and 48 h post-CLP, as indicated. Survival comparisons between the groups was done by a log-rank test. *N* = 10 per group. MEDI3622 + ertapenem vs. ertapenem, *p* < 0.0001. Ertapenem vs. saline, *p* = 0.12. (**B**) Sepsis severity for MEDI3622 + ertapenem and ertapenem-treated mice was assessed 12 h post-CLP. *N* = 10 mice in each group, data presented as mean ± SD, *** *p* < 0.001. (**C**) Neutrophil numbers were determined in the peritoneal lavage of MEDI3622 + ertapenem and ertapenem-treated mice 24 h post-CLP. *N* = at least 4 mice in each group, data presented as mean ± SD, * *p* < 0.05.

Prompt antibiotic intervention is a standard therapeutic approach for the management of sepsis patients, though its efficacy is highly variable [25]. Therefore, we introduced a broad-spectrum antibiotic in combination with MEDI3622 and assessed the effects on sepsis pathogenesis. Ertapenem has been used to manage sepsis in human subjects [26], and it has been used in the mouse CLP sepsis model [27]. Using a previously established dosing strategy for mice [27], we examined ertapenem administered at 75 mg/kg intraperitoneally at 6, 24, and 48 h post-CLP. We found that antibiotic treatment alone had a marginal though not significant effect on sepsis survival (Figure 2A), similar to other studies [28]. Interesting is that when MEDI3622 was administered pre-CLP followed by ertapenem post-CLP, survival was dramatically increased compared to mice that received ertapenem alone (Figure 2A). For example, at five days post-CLP, mice treated with MEDI3622 plus ertapenem demonstrated 10% mortality, whereas mice treated with ertapenem alone demonstrated 95% mortality (Figure 2A). Consistent with their improved survival, the sepsis severity score for MEDI3622 plus ertapenem-treated mice was significantly lower than the sepsis score for mice that received only ertapenem (Figure 2B). We again examined peritoneal levels of neutrophils at 24 h post-CLP for both treatment groups, which was significantly increased in the MEDI3622 plus ertapenem-treated mice (Figure 2C). 

**Figure 3:**
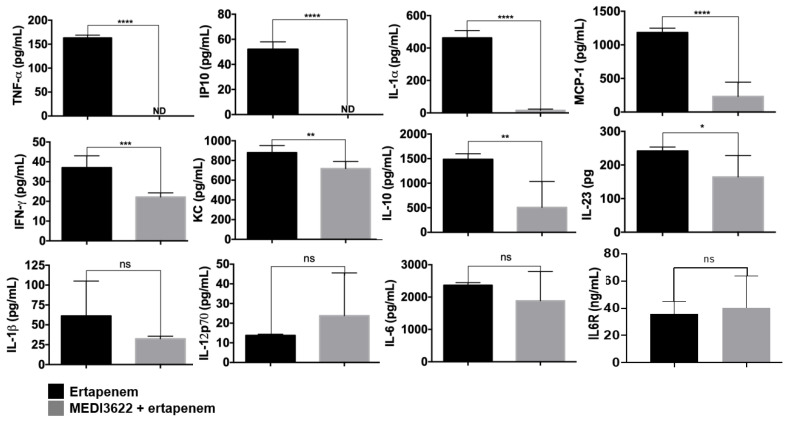
A combination of MEDI3622 and antibiotic treatment alters the plasma levels of various cytokines and chemokines. Mice were treated as described in Figure 2. Plasma levels of the indicated analytes were quantified 24 h post-CLP. *N* = at least 4 mice per group, ND = not detectable, data presented as mean ± SD, * *p* < 0.05, ** *p* < 0.01, *** *p* < 0.001, **** *p* < 0.0001.

Sepsis pathogenesis increases with higher circulating levels of proinflammatory cytokines [29]. At 24 h post-CLP, we examined the plasma levels of an array of inflammatory factors. For the mice treated with MEDI3622 plus ertapenem or ertapenem alone, we observed a considerably different profile of cytokines and chemokines. TNFα was undetectable in the plasma of MEDI3622 and ertapenem-treated mice (Figure 3), which is consistent with this cytokine being a well-established substrate of ADAM17 [10], as its original name indicates (TNFα converting enzyme or TACE) [30]. High levels of TNFα can impair vascular function and neutrophil migration during sepsis [31,32]. This cytokine is also critical for host defense and blocking its activity during sepsis, as well as other anti-cytokine strategies, is akin to a “double-edged sword” in that inflammation as well as the immune response are being blocked [33]. The clinical benefits of blocking TNFα activity during sepsis have varied depending on the approach, ranging from harmful to limited benefits [33]. Blocking ADAM17, however, only prevents the conversion of membrane TNFα to its soluble form and does not block its activity. Interesting is that directly blocking the shedding of TNFα has been shown to reduce systemic inflammation but enhance localized antimicrobial activity, and to be protective in a mouse model of CLP-induced sepsis [34]. 

The plasma levels of the chemokines Interferon γ-induced Protein 10 kDa (IP-10) and Monocyte Chemoattractant Protein-1 (MCP-1) and the cytokine IL-1⍺ were also dramatically decreased in MEDI3622 plus ertapenem-treated mice, and a significant reduction in Interferon-γ (IFNγ), Keratinocyte Chemoattractant (KC), IL-10 and IL-23 was observed as well (Figure 3). The chemokine receptor for MCP-1 is C-C chemokine receptor type 2 (CCR2) and its expression and activity are induced in circulating neutrophils during sepsis [35]. This has been reported to promote their migration to remote organs and cause tissue damage [36]. Hence, lower MCP-1 levels upon MEDI3622 and antibiotic treatment might diminish neutrophil sequestration in distal organs and damage at these sites. The reduced levels of IL-10 is also notable considering its immunosuppressive role [37]. Its production has been reported to be increased in sepsis patients and this was associated with a poorer prognosis [38]. Other than TNFα, the cytokines and chemokines that were significantly reduced in MEDI3622 plus ertapenem-treated mice during sepsis are not ADAM17 substrates. Their reduced levels may be the result of downstream effects of greatly reduced TNFα release and/or the result of reduced bacterial levels due the presence of ertapenem. We did not observe a significant change in the plasma levels of IL-1β and IL-12p70, or IL-6 in the two treatment groups 24 h post-CLP (Figure 3). However, their levels might be significantly different at other time points post-CLP. An interesting aspect of IL-6 signaling is that its receptor is a well-described ADAM17 substrate [10]. The cleaved form of the IL-6R binds to secreted IL-6 and increases its activity via trans-signaling through the ubiquitously expressed glycoprotein 130 [39]. We also examine the plasma levels of IL-6R in mice treated with ertapenem +/− MEDI3622 but did not observe a significant reduction at 24 h post-CLP (Figure 3). Plasma levels of IL-6R were also found not to be different between ADAM17 gene-targeted mice and control mice in other acute inflammation models [40], indicating that other sheddases are involved in the production of soluble IL-6R.

### 2.3. MEDI3622 Administration Post-CLP Prolongs Survival

**Figure 4:**
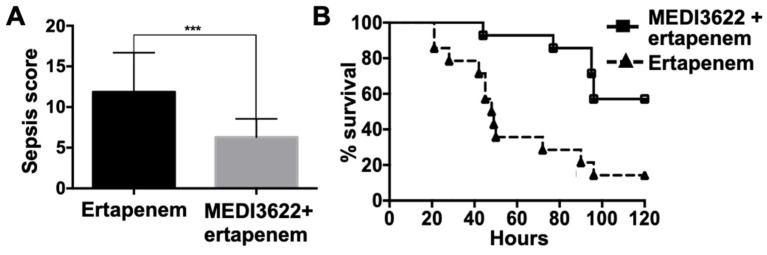
Effects of a combination of MEDI3622 and antibiotic treatment post-sepsis induction. (**A**) Mice were subjected to CLP (18G needle) and *ip* administered MEDI3622 (15 mg/kg) 6 h post-CLP and ertapenem (75 mg/kg) *ip* at 6, 24, and 48 h post-CLP or ertapenem (75 mg/kg) *ip* at 6, 24, and 48 h post-CLP. Sepsis severity was assessed at 12 h post-CLP. *N* = 14 mice per group, data presented as mean ± SD, *** *p* < 0.001. (**B**) Survival comparison between the two treatment groups was done by a log-rank test. *N* = 14 mice per group, *p* < 0.001.

Next, we evaluated the effects of administering both MEDI3622 and ertapenem post-CLP. In these experiments, MEDI3622 administration was performed intraperitoneally 6 h post-CLP along with ertapenem at 6, 24, and 48 h post-CLP. Again, MEDI3622 and ertapenem-treated mice exhibited a significantly lower sepsis score and mortality compared to mice treated with ertapenem alone (Figure 4A,B). We did not examine MEDI3622 treatment alone post-CLP. When MEDI3622 was administered alone prior to sepsis induction, the survival rate was lower than when MEDI3622 was administered in combination with ertapenem (Figure 1B). We speculate that this would also be the case after sepsis induction. Other limitations of our proof-of-concept study are that we only examined severe sepsis in which 100% of the control mice died (Figure 1B) and that MEDI3622 is a human mAb, which limits multi-dosing in immunocompetent mice. In further investigations, it will be interesting to convert MEDI3622 to a mouse chimeric antibody to reduce immunogenicity and to utilize it for multiple dosing, especially with a less severe sepsis model to examine its effects during early and prolonged sepsis, on the myeloid and lymphoid compartments, and on susceptibility to secondary infections.

In summary, ADAM17 functions as a regulatory checkpoint of various cytokines, receptors, and adhesion molecule, controlling their soluble levels and cell surface density [9]. We have previously demonstrated that gene-targeting of ADAM17 in all leukocytes decreased mortality during severe polymicrobial sepsis, which was associated with reduced levels of circulating cytokines and chemokines, increased neutrophil recruitment, and increased bacterial clearance [15,16,17]. In our current study, we report a similar host response when systemically targeting ADAM17 with a function-blocking mAb, which is a more clinically applicable approach. Of further clinical relevance is that MEDI3622 administration was combined with a conventional antibiotic. This resulted in a markedly enhanced survival benefit. Thus, the immune-modulatory therapy and antibiotic appeared to have synergistic effects in augmenting the early host response. Previous studies have eloquently established that infiltrating neutrophils must achieve a critical concentration for effective bacterial clearance [6,7]. We propose that blocking ADAM17 along with antibiotic administration will reduce leukocyte paralysis and bacterial viability, respectively, in turn decreasing the threshold and time to achieve a critical neutrophil concentration for sustained bactericidal activity. This combination therapy in clinical settings may be efficacious for sepsis patients and when used prophylactically for surgical patients at high risk of post-traumatic and post-operative sepsis. 

## 3. Materials and Methods

### 3.1. Animals

Mice were housed in a specific pathogen-free facility, and all procedures performed were done in accordance with protocols approved by the Institutional Animal Care and Use Committee of the University of Minnesota [1612-34435A]. Weight matched (26–30 g) C57BL/6J mice were utilized in this study. Induction of polymicrobial sepsis by CLP was performed as we have previously described [16]. A modification in the procedure was that the cecum was punctured with a sterile 18G needle through-and-through twice, resulting in four perforations. Mice were examined post-CLP using a sepsis scoring system that assessed eight individual criteria, namely appearance, consciousness, activity, response to stimulus, eyes, respiration rate, respiration quality, and diarrhea [16]. The sepsis survival rate was assessed by monitoring mice for 5 days after sepsis induction.

### 3.2. Administration of MEDI3622 and Antibiotics

MEDI3622 is a human IgG1 anti-ADAM17 mAb that blocks its function [21,22]. The pharmacokinetic and pharmacodynamic assessment of MEDI3622 has been performed in mice and the mAb demonstrated no significant adverse effects after continuous administration [21]. We observed no alterations in the health of mice administered MEDI3622 alone (intraperitoneally at 15 mg/kg) or saline carrier, which was based on behavior, gross anatomy of internal organs, including the spleen and liver, and our sepsis scoring system.

MEDI3622 was administered intraperitoneally at a dosage of 15 mg/kg. Ertapenem, a broad-spectrum antibiotic with antimicrobial activity against Gram-positive and Gram-negative bacteria [41], was administered intraperitoneally at the dose of 75 mg/kg at 6, 24, and 48 h after sepsis induction, based on a previously established dosing strategy [27].

### 3.3. Quantification of Plasma Analytes

Plasma samples were isolated and immediately stored at −80 °C. Plasma cytokine levels were determined by a cytokine multiplex assay, as we have described previously [16]. A mouse inflammation multiplex kit was used to detect 11 cytokines (TNF⍺, IP-10, IL-1⍺, IL-1β, IL-6, IL-10, IL-12p70, IL-23p19, MCP-1, IFNγ and KC) were performed according to the manufacturer’s protocol (Antigenix America, Huntington Station, NY, USA). Soluble mouse IL-6R levels were quantified using RayPlex Beads according to the manufacturer’s protocol (RayBiotech, Peachtree Corners, GA, USA). Mean fluorescent intensities (MFI) of the samples and standards were determined using a FACS Celesta instrument (BD Biosciences, San Jose, CA, USA). Analyte concentrations in the samples were a function of MFI, determined using the manufacturer’s recommended standard curves and dilution factors. Sample dilutions ranged from undiluted to 1/50 and MFIs for all cytokines fell within the range of the standard curve, except for TNFα and IP-10, which were below the standard curve for some samples. 

### 3.4. Enumeration of Blood and Peritoneal Fluid Levels of Neutrophils and Bacteria

The absolute number of neutrophils were determined in freshly harvested blood and peritoneal lavage of mice. Briefly, 100 μL of blood or peritoneal lavage was stained for neutrophils and the absolute counting was performed on a flow cytometer using a bead counting method (AccuCheck, Thermo Scientific, Waltham, MA, USA) as we have previously described [12,16]. Antibodies used to identify neutrophils included anti-Mac-1 (M1/70), anti-F4/80 (BM8) and anti-Ly-6G (RB6-8C5) (BioLegend, San Diego, CA, USA). The appropriate isotype-matched negative control antibodies were purchased from the same source. Bacterial counts were determined by plating 10-fold serial dilutions of blood or peritoneal lavage fluid on brain heart infusion agar plate (Becton Dickinson, Sparks, MD, USA), as previously described [16]. 

### 3.5. Statistical Analysis

The data analysis was performed using GraphPad Prism 7.0 (GraphPad Software, San Diego, CA, USA). The means of various parameters like bacterial load, neutrophil count, cytokine release, and sepsis severity in different groups of mice were compared through ANOVA and Student’s *t*-test. Survival comparisons between the various groups of mice were done by a log-rank (Mantel–Cox) test.
